# Microbial communities related to biodegradation of dispersed Macondo oil at low seawater temperature with Norwegian coastal seawater

**DOI:** 10.1111/1751-7915.12303

**Published:** 2015-07-16

**Authors:** Odd G Brakstad, Mimmi Throne-Holst, Roman Netzer, Donald M Stoeckel, Ronald M Atlas

**Affiliations:** 1 Department Applied Environmental Biology and Chemistry, SINTEF Materials and Chemistry, Environmental TechnologyTrondheim, N-7465, Norway; 2 Battelle Memorial InstituteColumbus, OH, 43201, USA; 3 University of LouisvilleLouisville, KY, 40292, USA

## Abstract

The Deepwater Horizon (DWH) accident in 2010 created a deepwater plume of small oil droplets from a deepwater well in the Mississippi Canyon lease block 252 (‘Macondo oil’). A novel laboratory system was used in the current study to investigate biodegradation of Macondo oil dispersions (10 μm or 30 μm median droplet sizes) at low oil concentrations (2 mg l^−1^) in coastal Norwegian seawater at a temperature of 4–5°C. Whole metagenome analyses showed that oil biodegradation was associated with the successive increased abundances of Gammaproteobacteria, while Alphaproteobacteria (*Pelagibacter*) became dominant at the end of the experiment. *Colwellia* and Oceanospirillales were related to n-alkane biodegradation, while particularly *Cycloclasticus* and *Marinobacter* were associated with degradation of aromatic hydrocarbons (HCs). The larger oil droplet dispersions resulted in delayed sequential changes of Oceanospirillales and *Cycloclasticus*, related with slower degradation of alkanes and aromatic HCs. The bacterial successions associated with oil biodegradation showed both similarities and differences when compared with the results from DWH field samples and laboratory studies performed with deepwater from the Gulf of Mexico.

## Introduction

After marine oil spills indigenous oil-degrading bacteria bloom and increase their abundances dramatically (Braddock *et al*., 1995; Yakimov *et al*., 2007), most of these belonging to the classes Alphaproteobacteria or Gammaproteobacteria (Yakimov *et al*., 2007). While both alpha- and gammaproteobacterial oil degraders are abundant during oil biodegradation in temperate seawater, Gammaproteobacteria become enriched at low seawater temperatures (Brakstad and Lødeng, 2005; Deppe *et al*., 2005; Coulon *et al*., 2007; Hazen *et al*., 2010; Bælum *et al*., 2012; Dubinsky *et al*., 2013). In marine oil spills aliphatic hydrocarbon (HC)-degrading bacteria like *Alcanivorax* are often succeeded by other bacterial populations like *Cycloclasticus* that attack the more slowly biodegradable oil compounds like polycyclic aromatic HCs (PAH) (Kasai *et al*., 2002a,b). Several HC degraders, including *Colwellia*, *Marinobacterium*, *Marinomonas*, *Glaciecola* and *Pseudoalteromonas*, have previously been associated with cold marine environments (Yakimov *et al*., 2004a; Deppe *et al*., 2005; Brakstad *et al*., 2008).

During the Deepwater Horizon (DWH) oil spill, to which the dispersant Corexit 9500 had been added at the wellhead, a deepwater plume (900–1300 m in depth) with finely dispersed oil at low concentrations and low seawater temperatures (4–6°C) was reported in several studies (Camilli *et al*., 2010; Reddy *et al*., 2012). Simulation of the plume trajectories indicated that oil droplets with diameters between 10 and 50 μm were abundant in this zone and were transported horizontally, while droplets larger than 90 μm rapidly rose to the surface (North *et al*., 2011). During the spill, elevated population levels of indigenous deep-sea bacteria associated with HC biodegradation (Oceanospirillales, *Cycloclasticus*, *Colwellia*, *Methylophaga*) were found in association with the deepwater plume (Hazen *et al*., 2010; Valentine *et al*., 2010; Redmond and Valentine, 2012; Dubinsky *et al*., 2013; Yang *et al*., 2014). Zones of oxygen anomalies, indicative of oil biodegradation, were also associated with the dispersed oil in the deepwater, resulting in a slight reduction of oxygen saturation from 67% outside to 59% inside the plume (Hazen *et al*., 2010).

Both field observations and laboratory experiments indicated short biodegradation half-lives for nC_13_-nC_26_ alkanes, ranging from 1 to 8 days (Hazen *et al*., 2010). Water samples associated with the plume had lower microbial diversities than samples outside the plume (Hazen *et al*., 2010; Mason *et al*., 2012) and were enriched with a variety of metabolic genes involved in both aerobic and anaerobic HC degradation, including genes for motility, chemotaxis and aliphatic HC degradation, while genes encoding aromatic HC degradation were expressed only at low levels (Lu *et al*., 2012; Mason *et al*., 2012). Laboratory studies showed that mixing of the Macondo oil with the dispersant resulted in faster oil biodegradation (Hazen *et al*., 2010; Bælum *et al*., 2012). A model was suggested for the deep sea biodegradation of HCs with sequential and pulsed propagation of bacteria degrading specific HCs, including n-alkanes, aromatics and C_1_–C_3_ gas components (Valentine *et al*., 2012). Bacterial respiration of gas compounds (mainly propane and ethane) was suggested to accelerate the response of HC-degrading bacteria (Valentine *et al*., 2010), while methane was oxidized by methanotrophic bacteria, but at a slower rate than for propane (Kessler *et al*., 2011).

The aim of this study was to determine the relative abundances of microbes related to biodegradation of specific oil compound groups, as well as the effect of oil droplet size on biodegradation, using Macondo oil in natural seawater at a temperature relevant for the Gulf of Mexico (GoM) deepwater. We used a novel experimental system, generating dispersed oil at two different droplet size distributions in an oil droplet generator, with incubation of the dispersions in a slowly rotating carousel system (Brakstad *et al*., 2015). The experiment was performed with permanently cold seawater from a Norwegian fjord, and results were compared with relevant field data from the DWH spill.

## Results and discussions

The biodegradation experiment with the Macondo oil premixed with Corexit 9500 [dispersant-to-oil ratio (DOR) 1:100] was performed in a slowly rotating carousel system after generation of dispersions with 10 μm or 30 μm median oil droplet sizes and nominal concentrations of 2 mg l^−1^. These dispersions were prepared with seawater pre-acclimated for 6 days (5°C), and with low oil concentration (0.2 mg l^−1^ of Macondo oil). The experiment was performed at 5°C for 64 days (Brakstad *et al*., 2015). Consistency of oil droplet size distributions was measured by Coulter counter during the biodegradation period. Droplet sizes, measured by Coulter counter, were maintained in the 10 μm dispersions, while the 30 μm dispersions in unfiltered seawater showed a rapid decline in droplet size between days 0 and 8. However, the 30 μm median oil droplet diameter was maintained in the sterile dispersions, as recently reported (Brakstad *et al*., 2015). Although no addition of nutrients or oxygenation was performed during the experimental period, we did not experience oxygen or nutrient (NO_3_^−^-N/NO_2_^−^-N, NH_4_^+^-N, *o*-PO_4_^2−^-P) depletion (Brakstad *et al*., 2015).

### Microbial concentrations

**Figure 1:**
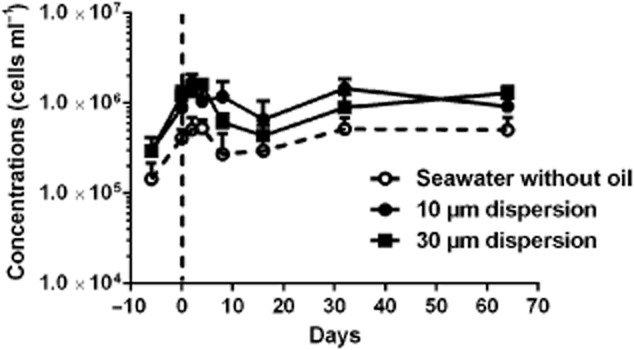
Cell concentrations determined by epifluorescence microscopy 4′,6-diamidino-2-phenylindole (DAPI) in dispersions and seawater without oil. In addition to samples from the biodegradation experiment (days 0–64), seawater and dispersions (0.2 mg l^−1^ oil) for acclimation are shown on the left of the vertical line (day −6).

Bacterial counts of the source seawater showed cell concentrations of 1.44 ± 0.77–2.95 ± 1.19 × 10^5^ cells ml^−1^ (Fig. 1); these concentrations are similar to results from previous samples of the same water source (Brakstad *et al*., 2004). During the biodegradation period average concentrations of 3.97 × 10^5^ (range 1.44 × 10^5^–5.25 × 10^5^), 10.2 × 10^5^ (range 2.95 × 10^5^–16.8 × 10^5^) and 9.61 × 10^5^ (range 2.95 × 10^5^–16.0 × 10^5^) cells ml^−1^ were measured in seawater without oil, 10 μm dispersions and 30 μm dispersions respectively (Fig. 1). During the 6-day acclimation period increased cell concentrations were measured both in source seawater and in the 10 μm dispersion used for acclimation (Fig. 1). From the start to the end of the biodegradation experiment, cell counts increased by a factor of 1.24 in the seawater without oil and by 1.03–1.07 in the dispersions. Cell concentrations in the 10 μm and 30 μm dispersions were higher than in the seawater without oil, with average factors of 2.7 and 2.4, in favour of the 10 and 30 μm dispersions respectively. The concentrations became slightly higher in the 10 than the 30 μm dispersions, from 16 to 64 days of the experiment. The bacterial concentrations in the Norwegian water used in this experiment were higher than in samples collected from the GoM deepwater during the ongoing spill, which ranged from 1 to 5.5 × 10^4^ cells ml^−1^ (Atlas and Hazen, 2011; Bælum *et al*., 2012).

### Microbial communities

A total of 17 DNA extracts were subject to metagenome sequence analyses, including DNA extracts from four samples of seawater without oil added, seven oil dispersions of 10 μm and six dispersions of 30 μm droplet size. Initial analyses indicated high abundances of *Pelagibacter* sp., *Colwellia psychrerythraea*, *Pseudomonas syringae*, *Pseudomonas fluorescens* and *Xanthomonas translucens* (not shown). A confirmatory reference alignment analysis was performed for *Pelagibacter* sp., *C. psychrerythraea*, *P. syringae*, *P. fluorescens*, *X. translucens* and PhiX174 (control) full-length genomes. Sequence data for *Pelagibacter* sp., *C. psychrerythraea* and the control PhiX174 revealed over 95% coverage over the full length reference genomes. Reads matching *P. syringae*, *P. fluorescens* and *X. translucens* in reference alignments revealed < 1% coverage and aligned sequences matched only a few, limited genetic regions; therefore, these organisms were removed from subsequent analysis.

**Figure 2:**
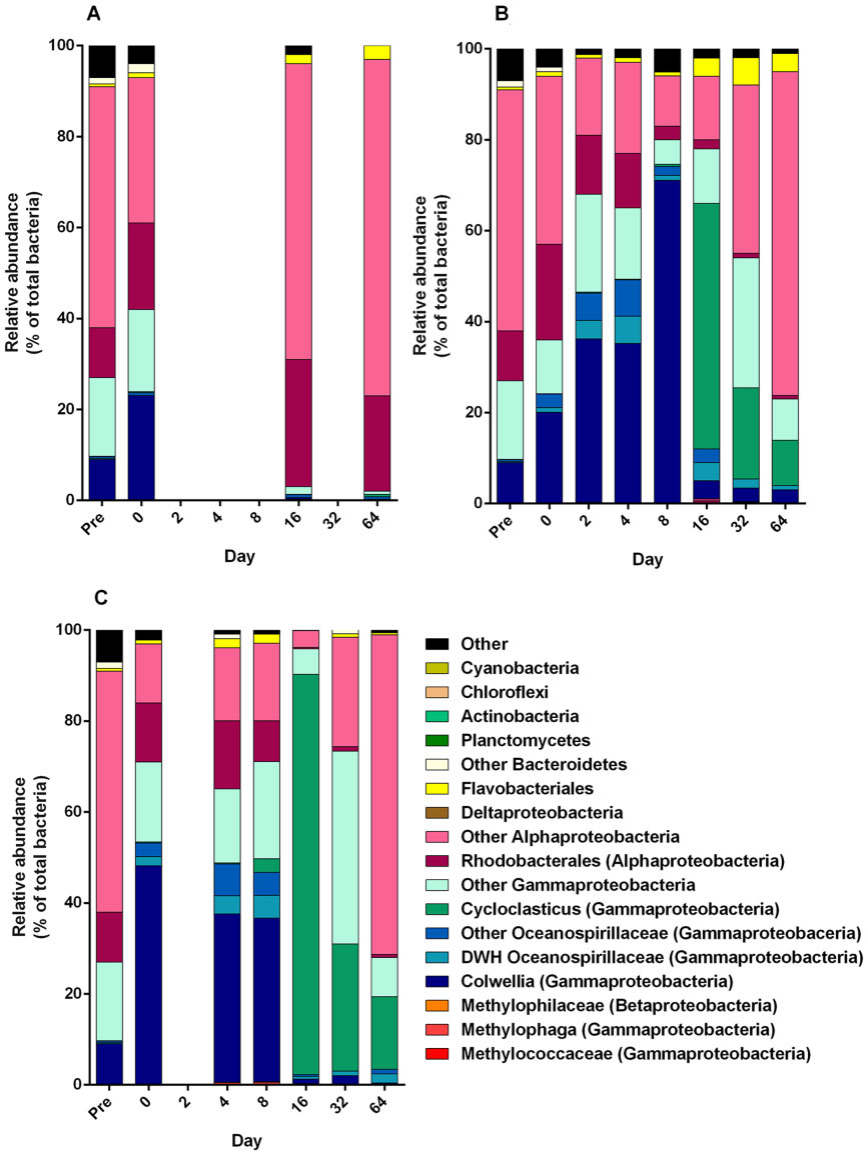
Relative abundances of bacterial groups in seawater without oil (A), 10 μm dispersions (B) and 30 μm dispersions (C), classified as described by Redmond and Valentine (2012). The results are shown in seawater used for pre-acclimation 6 days prior to start of biodegradation (Pre), and in seawater and dispersions collected at the start of the experiment (0), and after 2–64 days of biodegradation.

The bacterial communities in the seawater without oil were dominated by Alphaproteobacteria, with 51–95% abundance, while Gammaproteobacteria showed 2–42% abundances (Fig. 2A and Fig. S1). However, in the oil dispersions the Gammaproteobacteria became rapidly predominant after 2 to 32 days of biodegradation, with 54–80% and 65–96% abundances in the 10 μm and 30 μm dispersions respectively. The Gammaproteobacteria abundances peaked after day 8 in the 10 μm dispersions, and after day 16 in the 30 μm dispersions. Thereafter the abundances decreased in the dispersions, and after 64 days the Alphaproteobacteria became predominant in both dispersions (72% and 71% abundances in the 10 μm and 30 μm dispersions, respectively), similarly to the seawater without oil (Fig S1). Typically, oil biodegradation in cold marine seawater is associated with abundances of Gammaproteobacteria, shown in both laboratory and field studies (e.g. Yakimov *et al*., 2004a; Deppe *et al*., 2005; Gerdes *et al*., 2005; Brakstad and Bonaunet, 2006; Hazen *et al*., 2010; Redmond and Valentine, 2012; Dubinsky *et al*., 2013).

For the comparison of the microbial communities in our study with field results from the DWH oil spill, we used a grouping system recently described for DWH deepwater field samples (Redmond and Valentine, 2012). In the seawater without oil, the Alphaproteobacteria were distributed between Rhodobacterales (11–28% abundances) and ‘other Alphaproteobacteria’ (32–74% abundances), as shown in Fig. 2A. The ‘other Alphaproteobacteria’ were dominated by *Pelagibacter* of the SAR 11 clade (21–63% abundances). *Pelagibacter* is accepted as the most abundant group of heterotrophic bacteria in the oceans, and represent approximately one-quarter of all 16S ribosomal RNA (rRNA) genes identified in clone libraries from marine environments (Morris *et al*., 2002). High abundance of this genus in the seawater without oil was therefore expected. The groups of Gammaproteobacteria in the seawater included *Colwellia* (0–23% abundances) and ‘other Gammaproteobacteria’ (1–18% abundances), including *Marinobacter* and unclassified Gammaproteobacteria, appearing at the start of the experiment (Fig. 2A).

The increasing enrichments of Gammaproteobacteria in the oil dispersions followed a succession, with *Colwellia* and Oceanospirillales being abundant during the first 8 days, with subsequent predominances of *Cycloclasticus* after 16–64 days in both dispersions (Fig. 2B and C). Abundances of *Colwellia* increased between days 0 and 8 from 20% to 71% in the 10 μm dispersions and were maintained between 36% and 40% in the 30 μm dispersions. From 16 to 64 days *Collwellia* was reduced to ≤ 4% abundance in both dispersions. Also Oceanospirillales increased during the early phase of the experiment, from 4% to 14% and from 5% to 11% between day 0 and 4 in the 10 μm and 30 μm dispersions respectively. The abundances of Oceanospirillales then declined during the rest of the period. The group described as DWH Oceanospirillales (Fig. 2) refers to uncultivated Oceanospirillales observed in DWH deepwater plume (Hazen *et. al*., 2010; Mason *et al*., 2012; Redmond and Valentine, 2012) and accounted for 25–100% of the Oceanospirillales in the dispersions. Typically, the DWH Oceanospirillales emerged later than ‘other Oceanospirillales’ in the dispersions. The reductions of both groups of Oceanospirillales came later in the 30 μm than the 10 μm dispersions (Fig. 2B and C). The genera associated with the Oceanospirillales included *Bermanella* and *Thalassolituus*, which also are some of the closed cultured relatives to the DWH Oceanospirillales (Yang *et al*., 2014).

Following the reduced abundances of *Colwellia* and Oceanospirillales, increased levels of *Cycloclasticus* appeared. *Cycloclasticus* constituted 54% and 88% of the bacterial populations in the 10 μm and 30 μm dispersions after 16 days, with subsequent declines to 10% and 16% in the two dispersions after 64 days, and were closely affiliated to the pyrene-degrading *Cycloclasticus* sp. strain P1 (Lai *et al*., 2012). As with Oceanospirillales, the decline of *Cycloclasticus* was slower in the 30 μm than the 10 μm dispersions. At the end of the experimental period (32 and 64 days), additional genera of Gammaproteobacteria became abundant, shown as ‘other Gammaproteobacteria’ (Fig. 2B and C), mainly affiliated with *Marinobacter* (20% and 29% abundances in the 10 μm and 30 μm dispersions respectively). In contrast to the late abundance of *Marinobacter* in oil dispersions, this genus appeared early in seawater without oil. The predominances of ‘other Alphaproteobacteria’ in both dispersions at the end of the experiment (70–71% abundances) were associated with *Candidatus Pelagibacter* in both dispersions. The dispersed oil therefore resulted in stimulation of mainly Gammaproteobacteria, following a succession in three steps, with an early phase of *Colwellia* and Oceanospirillales, a mid-phase of *Cycloclasticus* and a late phase of mainly *Candidatus Pelagibacter*, and with contributions of *Marinobacter*. The differences in succession patterns between 10 μm and 30 μm dispersions were mainly related to the slower decline of some of the bacterial groups after their peak abundances, including Oceanospirillales and *Cycloclasticus*.

The abundant bacteria in our laboratory study with Macondo oil dispersions in Norwegian coastal seawater showed some similarity to results from the GoM field samples of the deepwater plume during the DWH spill. The field samples from the DWH deepwater plume were also dominated by a few Gammaproteobacteria, including Oceanospirillales, *Colwellia* and *Cycloclasticus*, with an early increase in the abundances of Oceanospirillales, followed by later appearances of *Cycloclasticus* (Hazen *et al*., 2010; Valentine *et al*., 2010; Redmond and Valentine, 2012; Dubinsky *et al*., 2013). The genera *Bermanella* and *Thalassolituus*, which were the closest relatives the Oceanospirillales sequences in our study, were also associated with the DWH Oceanospirillales (Yang *et al*., 2014), and *Bermanella* has also been associated with natural marine crude oil seeps (Hawley *et al*., 2014). Most of the *Colwellia* sequences in our study were related to *C. psychrerythraea*, in agreement with the *Colwellia* sequences retrieved from the DWH deepwater plume (Mason *et al*., 2014). *Marinobacter*, which appeared late in the experiment with Norwegian seawater, was detected both in DWH deepwater plume and surface water (Hazen *et al*., 2010; Kostka *et al*., 2011), mainly after the spill period in September/October 2010 (Gutierrez *et al*., 2013). In accordance with our results, increased abundances of SAR11 were measured in the plume after well shut in (Yang *et al*., 2014).

However, the bacterial successions in the GoM field samples also differed from our results. The DWH field samples showed much higher abundances of Oceanospirillales in early plume samples than in our studies (Hazen *et al*., 2010; Redmond and Valentine, 2012; Dubinsky *et. al*., 2013). Further, these field studies showed late abundances of *Colwellia*, similar to the *Cycloclasticus* (Redmond and Valentine, 2012; Dubinsky *et al*., 2013). In addition, the field samples were enriched with *Pseudomonas* and *Pseudoalteromonas* (Redmond and Valentine, 2012; Dubinsky *et al*., 2013).

Laboratory studies of Macondo oil biodegradation with GoM deepwater at 4–5°C showed a relatively late abundance of *Colwelliaceae* compared with our results (Bælum *et al*., 2012; Wang *et al*., 2014). However, early abundances of Oceanospirillales in these studies (5–10%) were more in agreement with our results than with the field data (Bælum *et al*., 2012; Wang *et al*., 2014). These differences between GoM deepwater and Norwegian shallow water are expected, due to different geographical origin, depths and environmental conditions. The continuous exposure of oil from natural seepages in the GoM may be of particular importance for the presence of oil-degrading bacterial communities in the GoM (e.g. Smith *et al*., 2014).

Results from the DWH deepwater plume also showed methanotrophs and methylotrophs (*Methylomonas*, *Methylopha*ga), which were associated with methane oxidation and oil compound biodegradation during the DWH oil release (Valentine *et al*., 2010; Dubinsky *et al*., 2013). Methylotropic bacteria were only measured at ≤ 1% abundances in the dispersions (Fig. 2). This was not surprising, since C_1_ substrates like methane were not expected to be present in the dispersions studied here. However, we have previously detected *Methylophaga* during oil biodegradation with the same seawater source as used here (Brakstad *et al*., 2004).

Microbial diversities estimated by Shannon–Wiener diversity indexes did not differ between communities in seawater without oil and dispersions (Table S1), and the diversities were reduced both in seawater and dispersions over time. Low diversity indices were associated with relative enrichment of abundant bacterial genera described above, in particular *Pelagibacter* and *Cycloclasticus*.

In addition to the whole genome analyses bacterial 16S rDNA denaturing gradient gel electrophoresis (DGGE) revealed banding pattern differences between oil dispersions and seawater, and similarities between the two dispersions, with a banding pattern changing in the oil dispersions between day 8 and 16, and with separate cluster of seawater with oil and dispersions (Fig. S2).

### Comparison of bacterial analyses and petroleum HC biodegradation

Biotransformation data from this experiment have recently been presented for 22 volatile and semivolatile oil compound groups (Brakstad *et al*., 2015), as defined by Reed and colleagues (2000), representing 70–80% of the oil according to true boiling point curve (Pasquini and Bueno, 2007). First-order biotransformation rates of both saturates and aromatic HCs were generally faster in the 10 μm than in the 30 μm dispersions, showing the importance of oil droplet size distribution for biodegradation (Brakstad *et al*., 2015). Depletion data of C_5_–C_36_ n-alkanes, decalines monoaromatic HCs and PAHs are shown in the Supporting Information as 1^st^ order biotransformation decay curves (Figs S3 and S4), and as rate coefficients and half-lives (Table S2). Depletion of all compound groups was mainly the result of biodegradation, since sterilized controls showed only small or minor depletion during the experimental period. Both rate coefficients and half-lives of the n-alkane and aromatic HC groups were significantly different between the 10 μm and 30 μm dispersions in unfiltered seawater (P < 0.05 by non-parametric *t*-test analyses).

Biotransformation of the alkane and aromatic oil compound groups, based on the rate coefficients, was compared with the appearances of abundant bacterial groups in the dispersions (Table 1). The n-alkanes were mostly biodegraded between days 0 and 8, although nC_22_-nC_36_ alkanes required 16 days before > 80% biotransformation was reached. This early period was related to the abundances of Oceanospirillales and *Colwellia*. In accordance with the generally slower biotransformation of the n-alkanes in the 30 μm than in the 10 μm dispersions, the abundances of especially Oceanospirillales were delayed in the larger oil-droplet dispersions (Table 1). The later emergence of DWH Oceanospirillales than ‘other Oceanospirillales’ may be related to different n-alkane preferences of the two groups, and single cell sequencing of DWH Oceanospirillales has revealed genes for both n-alkane and cycloalkane degradation (Mason *et al*., 2012). Several groups within the order Oceanospirillales are associated with n-alkane biodegradation, including members of the genus *Thalassolituus* reported in our study (Yakimov *et al*., 2004b). However, no information of alkane degradation exists for *Bermanella*, the other major Oceanospirillales genus in our study (Pinhassi *et al*., 2009). *Colwellia* may metabolize a variety of oil compounds, including gaseous alkanes, aromatics and alkane-derived intermediates (Valentine *et al*., 2010; Redmond and Valentine, 2012; Mason *et al*., 2012; Gutierrez *et al*., 2013). *Colwellia* has been associated with cold oil-polluted marine environments like Antarctic and Arctic seawater and ice (Yakimov *et al*., 2004a; Brakstad *et al*., 2008), and have also been detected in water injection systems of offshore oil platforms (Korenblum *et al*., 2010).

**Table 1:**
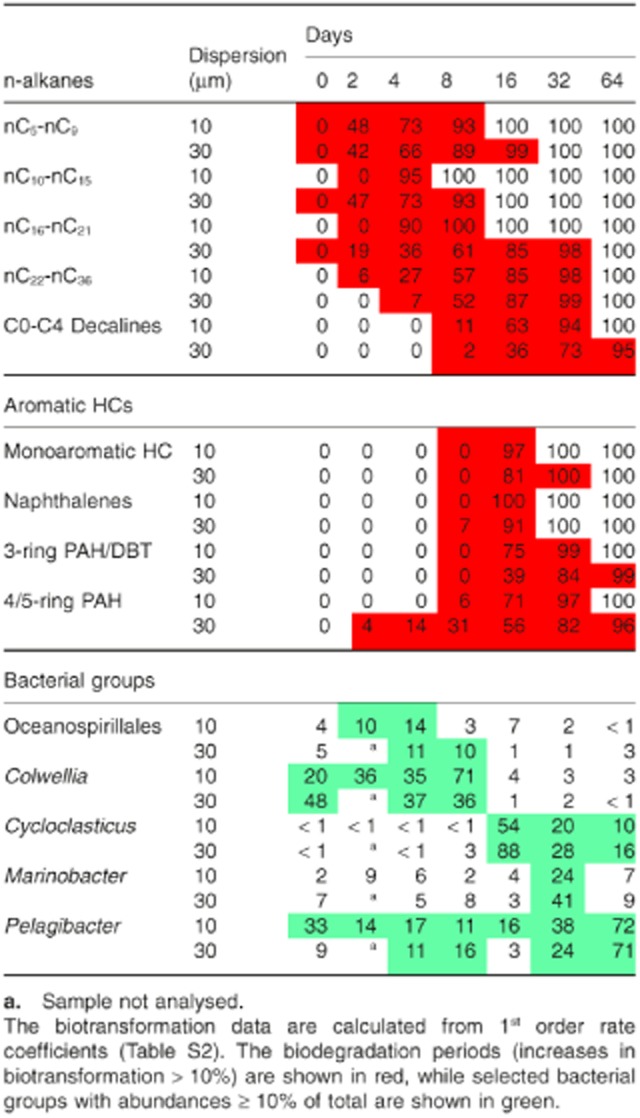
Comparison between biotransformation (%) of n-alkanes, aromatic hydrocarbons, and the relative abundances (% of total) of groups of Gammaproteobacteria and Alphaproteobacteria in the 10 μm and 30 μm oil dispersions

Biodegradation of aromatic HCs, both monoaromatics and PAHs, was slower than for n-alkanes, with the highest depletion between days 8 and 32. Nearly all monoaromatic HCs and naphthalenes were depleted between days 8 and 16 in the 10 μm dispersions, but also concentrations of three-ring and four/five-ring PAH were reduced by > 75% in the 10 μm dispersions during this period. Depletion of aromatic HCs was slower in the 30 μm than in the 10 μm dispersions and required 32 days to reach the same levels of depletion as in the 10 μm dispersions after 16 days. The predominance of *Cycloclasticus* was typically associated with biodegradation of aromatic HCs. These bacteria are well-known degraders of monoaromatics and PAH (Dyksterhouse *et al*., 1995; Geiselbrecht *et al*., 1998). High abundances of this genus remained longer in the 30 μm than in the 10 μm dispersions, in accordance with the longer degradation periods of the aromatic HCs in the larger droplet dispersions. Also *Marinobacter* is associated with biodegradation of alkanes, monoaromatic HCs and PAHs (Doumenq *et al*., 2001; Hedlund *et al*., 2001; Yakimov *et al*., 2007). The abundance of *Marinobacter* at day 32 is therefore likely related to aromatic HC degradation or long-chain or cyclic alkanes. Studies with water samples from the DWH spill also showed enrichment of *Cycloclasticus* and *Marinobacter* with naphthalene or phenanthrene as carbon sources (Gutierrez *et al*., 2013). The predominance of *Pelagibacter* at the end of biodegradation period may be part of a return to baseline conditions, in accordance with the high abundances of these groups in seawater without oil (Fig. 2A).

## Conclusions

This study presents the relative abundances of microbes associated with biodegradation of dispersed Macondo oil in coastal Norwegian seawater. The results from our studies showed relations between bacterial successions and oil compound biodegradation. Bacteria associated with oil compound biodegradation were dominated by Gammaproteobacteria. While Oceanospirillales and *Colwellia* were associated with n-alkane biodegradation, the later abundances of *Cycloclasticus* and *Marinobacter* corresponded with degradation of aromatic HCs, and possibly complex alkenes. The increasing abundances of the heterotrophic *Pelagibacter* at the end of the biodegradation period may also indicate the return to baseline conditions. The dispersions with the larger oil droplet distribution (30 μm) resulted in slower biodegradation of some of the oil compound groups than in the smaller droplet dispersion (10 μm), which also could be related to delayed abundances of some bacterial groups, mainly associated with biodegradation of aromatic HCs. Several of the bacterial groups enriched during biodegradation of the Macondo oil in the dispersions with Norwegian seawater were also associated with oil biodegradation in deepwater plume during the DWH accident and in laboratory studies with GoM deepwater. However, our study indicated different patterns of bacterial successions during biodegradation when compared with GoM water, reflecting differences between different geographical localities, depths and environmental conditions.

## Experimental procedures

### Oil dispersion generator and carousel system

An oil droplet generator and a carousel system were used in preparing and maintaining oil dispersions with defined droplet size distributions (Nordtug *et al*., 2011; Brakstad *et al*., 2015). By using seawater flow rates of 178 and 21 ml min^−1^, dispersions with median oil droplet sizes of 10 and 30 μm were generated (Fig. S5).

Dispersions were transferred to 2 l borosilicate flasks capped with teflon inserts (VWR International) which were completely filled and mounted on a carousel incubation system, as recently described (Brakstad *et al*., 2015). The carousel system rotated in clockwise direction at a velocity of 0.75 r.p.m. (Fig. S6).

### Experimental conditions

The seawater used (Trondheimsfjord, outside the harbor area of Trondheim, 63°26′N, 10°26′E) and the experimental conditions have recently been described (Brakstad *et al*., 2015). The oil used was unweathered Macondo oil collected directly from the subsea containment system at the MC252 wellhead by the production vessel Discoverer Enterprise on 22 May 2010. The oil was transferred from the production vessel to the Oil Barge Massachusetts (MASS oil). COREXIT 9500A (Nalco, Sugar Land, TX) dispersant was mixed with oil at a DOR of 1:100 at room temperature.

Briefly described, biodegradation of dispersed oil was performed in unfiltered pre-adapted seawater (0.2 mg l^−1^ of Macondo oil dispersions incubated on carousels at 4–5°C for 6 days) with median oil droplet sizes of 10 μm or 30 μm and nominal oil concentration of 2 mg l^−1^. Dispersions of 10 μm or 30 μm median oil droplet sizes and 2 mg l^−1^ nominal concentrations were also prepared in sterile-filtered (0.2 μm) seawater supplemented with HgCl_2_ (100 mg l^−1^). Flasks with unfiltered or sterilized dispersions were immediately mounted on the carousel at slow rotation (0.75 r.p.m.). Flasks with experimental blank samples (unfiltered seawater without oil) were also included in the experiment for background analyses and were pre-adapted without oil. Experiments were conducted at 4–5°C for up to 64 days, with sampling after 15 min (0 day samples), and after 2, 4, 8, 16, 32 or 64 days of incubation on the carousels. Samples of unfiltered dispersions were sacrificed for particle counting and chemical analyses (triplicate) or microbiological analyses (single samples), sterilized samples (single samples) for particle counting and chemical analyses, and seawater without oil for chemical and microbiological analyses (both single samples). In addition, a seawater sample was collected for microbiological analyses at the start of the pre-adaption period, 6 days prior to the start of the experiment.

### Microbiological analyses

Cell counts were performed by epifluorescence microscopy (Brakstad *et al*., 2008).

Both oil dispersions in seawater and seawater without oil (2 l) were filtered through 0.2 μm Sterivex polyvinylidene fluoride filters (Millipore, Billerica, MA, USA) and extracted by hot phenol–chloroform–isoamyl alcohol extraction (Sambrook and Russel, 2001).

Bacterial 16S rRNA gene sequences were amplified by polymerase chain reaction (PCR), and the PCR producte were analyses by DGGE as previously described (Brakstad and Bonaunet, 2006). Comparison of sample banding patterns was performed by unweighted pair group method with arithmetic mean, as dendrograms by the software Phoretix 1D (TotalLab Ltd., Newcastle, UK).

Metagenomic analyses were performed on 0.5–1 μg extracted DNA. Genomic DNA was fragmented by sonication (Covaris™ S220 Sonicator; Covaris, Inc., Woburn, MA) to approximately 300 base pairs (bp), and fragments were used to synthesize indexed sequencing libraries using the TruSeq DNA Sample Prep Kit V2 (Illumina, Inc., San Diego, CA), according to manufacturer's recommended protocol. Cluster generation was performed on the cBOT using the TruSeq PE Cluster Kit v3-cBot-HS (Illumina). Libraries were sequenced with an Illumina HiSeq 2000 at Nationwide Children's Hospital (NCH) Biomedical Genomics Core (Columbus, OH) using the TruSeq SBS Kit v3 reagents (Illumina) for paired end sequencing with read lengths of 100 bp (200 cycles). Primary analysis (image analysis and basecalling) was performed using HiSeq Control Software version 1.5.15.1 and Real Time Analysis version 1.13.48. Secondary analysis (demultiplexing) was performed using Illumina casava Software v1.6 on the NCH computed cluster. Sequence data were analysed by a custom in-house basic local alignment search tool (BLAST) (National Library of Medicine, Bethesda, MD) method to probe for organism identity. Quality-filtered sequence data (bases had a phred quality of ≥ 17, i.e. the probability of a correct base call was ∼ 98%) were converted to FASTA formats. Reads were searched against a database comprising > 1.4 million DNA genome sequences obtained from the *RefSeq* database v. 8/22/2013 (National Center for Biotechnology Information (NCBI), Bethesda, MD) from invertebrate organisms excluding plastids and plasmids. Top hit BLAST results were filtered for sequences with ≥ 97% identity and sequence length of ≥ 80 bps and post-processed by removal of top hits for any given taxa < 0.01% (1:10 000) of the total BLAST hits. Filtered BLAST results were classified to report the relative abundance of organisms identified in the sample.

On primary analysis the RefSeq database was determined to contain genome sequences of *P. syringae* and *X. translucens* that produced an abundance of false positive results in the BLAST analysis, confirmed by secondary reference alignment using CLC Genomics Workbench. A second bioinformatics analysis was therefore performed, in which *P. syringae*, *P. fluorescens* and *X. translucens* sequences had been omitted from the output data to provide reliable in-depth analysis of the data.

The sequences from this study were uploaded on the NCBI Sequence Read Archive (accession numbers SAMN03392593 – SAMN03392608).

### Chemical analyses and data treatment

Water samples were solvent-solvent extracted for measurements of target analytes by gas chromatography-mass spectrometry (GC-MS), including C_10_-C_36_ n-alkanes, two- to five-ring PAHs and the recalcitrant oil biomarker compound 17α(H),21β(H)-Hopane (30ab Hopane), as recently described (Brakstad *et al*., 2014). Volatile organic carbon (VOC) was determined by purge and trap GC-MS analyses (Brakstad *et al*., 2015). Aromatic VOC and SVOC target compounds included in GC-MS analyses are shown in Table S3.

Determined concentrations of target compound groups (Table S3) were normalized to 30ab Hopane (Prince *et al*., 1994), and biotransformation rates determined by non-linear regression analyses for calculation and 1^st^ order rate coefficients (k1) and half-lives, including lag phases, as recently described (Brakstad *et al*., 2015).

Column statistics were compared by Wilcoxon matched paired test in GraphPad Prism vs. 6.

### Other analyses

Oil droplet concentrations and size distributions were determined by Coulter counter measurements (Beckman Multisizer 4; Beckman Coulter Inc., Brea, CA) fitted with a 280 μm aperture, for measurement of droplets within a diameter range of 5.6–100 μm (Brakstad *et al*., 2015).

Bacterial community diversity was evaluated by calculation of Shannon–Wiener diversity index (Shannon and Weaver, 1963).

## Conflict of Interest

The authors have no conflict of interest to declare.

## Supporting Information

**Fig. S1.** Relative abundances of bacterial classes in seawater without oil, 10 μm dispersions and 30 μm dispersions. The results are shown in seawater used for pre-acclimation 6 days prior to start of biodegradation (Pre), and in seawater and dispersions collected at the start (0) and after 2–64 days of biodegradation. **Fig. S2.** Bacterial 16S rRNA PCR-DGGE of dispersions and seawater without oil. DGGE gels of samples at the start of acclimation (pre) and during the biodegradation period are shown in A, while comparison of samples as UPGMA dendrograms are shown in B. NC is negative control. **Fig. S3.** Depletion of nC_5_-nC_36_ alkanes in dispersions with unfiltered or sterilized (Ster) seawater. The depletion of SVOC n-alkanes (nC10-nC36 alkanes) was determined after normalizing targeted n-alkanes against the recalcitrant biomarker 17α(H),21β(H)-Hopane (30ab Hopane). The depletion in dispersions with unfiltered seawater is shown as nonlinear decay curves, determined by 1^st^ order rates with lag periods included. Data from non-linear regression analyses are shown in Table S3. **Fig. S4.** Depletion of aromatic hydrocarbons in dispersions with unfiltered or sterilized (Ster) seawater. The depletion of SVOC compounds (naphthalenes and PAH) was determined after normalizing targeted n-alkanes against the recalcitrant biomarker 17α(H),21β(H)-Hopane (30ab Hopane). The depletion in dispersions with unfiltered seawater is shown as nonlinear decay curves, determined by 1^st^ order rates with lag periods included. Data from non-linear regression analyses are shown in Table S3. **Fig. S5.** Average oil droplet size distributions generated in the oil dispersion generator at different seawater flow rates after premixing of the oil with Corexit 9500A. **Fig. S6.** Oil dispersion carousel with flasks mounted. Flasks are completely filled with oil dispersions, mounted on the carousel which is slowly rotated in clockwise direction at 0.75 r.p.m. **Table S1.** Bacterial diversity determined by Shannon–Wiener index measurements. **Table S2.** Kinetics data for groups of nC_5_-nC_36_ alkanes and aromatic compounds in 10 μm dispersions of Macondo oil determined by 1^st^ order rate approach with lag phases included. Half-lives were determined from rate coefficients and lag phases included. The goodness of fit of non-linear regressions was determined as R^2^. **Table S3.** Aromatic hydrocarbon target groups from GC-MS analyses of volatile and semivolatile compounds included in this study.
