# Supplementary material for: Microbial communities related to biodegradation of dispersed Macondo oil at low seawater temperature with Norwegian coastal seawater
Source: Microb Biotechnol. 2015 Jul 16;8(6):989–98. doi: 10.1111/1751-7915.12303 (PMC4621451; doi:10.1111/1751-7915.12303)
Supplement: Supplementary file 1 — Fig. S1. Relative abundances of bacterial classes in seawater without oil, 10 μm dispersions and 30 μm dispersions. The results are shown in seawater used for pre-acclimation 6 days prior to start of biodegradation (Pre), and in seawater and dispersions collected at the start (0) and after 2–64 days of biodegradation. Fig. S2. Bacterial 16S rRNA PCR-DGGE of dispersions and seawater without oil. DGGE gels of samples at the start of acclimation (pre) and during the biodegradation period are shown in A, while comparison of samples as UPGMA dendrograms are shown in B. NC is negative control. Fig. S3. Depletion of nC5-nC36 alkanes in dispersions with unfiltered or sterilized (Ster) seawater. The depletion of SVOC n-alkanes (nC10-nC36 alkanes) was determined after normalizing targeted n-alkanes against the recalcitrant biomarker 17α(H),21β(H)-Hopane (30ab Hopane). The depletion in dispersions with unfiltered seawater is shown as nonlinear decay curves, determined by 1st order rates with lag periods included. Data from non-linear regression analyses are shown in Table S3. Fig. S4. Depletion of aromatic hydrocarbons in dispersions with unfiltered or sterilized (Ster) seawater. The depletion of SVOC compounds (naphthalenes and PAH) was determined after normalizing targeted n-alkanes against the recalcitrant biomarker 17α(H),21β(H)-Hopane (30ab Hopane). The depletion in dispersions with unfiltered seawater is shown as nonlinear decay curves, determined by 1st order rates with lag periods included. Data from non-linear regression analyses are shown in Table S3. Fig. S5. Average oil droplet size distributions generated in the oil dispersion generator at different seawater flow rates after premixing of the oil with Corexit 9500A. Fig. S6. Oil dispersion carousel with flasks mounted. Flasks are completely filled with oil dispersions, mounted on the carousel which is slowly rotated in clockwise direction at 0.75 r.p.m. Table S1. Bacterial diversity determined by Shannon–Wiener ind [file mbt20008-0989-sd1.docx]

**Supporting Information**

Table S1. Bacterial diversity determined by Shannon-Wiener index measurements.

| **Incubation period (days)** | **Seawater** | **10 µm dispersions** | **30 µm dispersions** |
| --- | --- | --- | --- |
| -6 (pre-incubation) | 2.99 |  |  |
| 0 | 3.03 | 3.00 | 2.97 |
| 2 | ^A)^ | 3.27 | ^A)^ |
| 4 | ^A)^ | 3.30 | 3.38 |
| 8 | ^A)^ | 2.07 | 3.39 |
| 16 | 2.23 | 2.68 | 1.09 |
| 32 | 2.02 | 2.86 | 2.71 |
| 64 | ^A)^ | 1.48 | 2.12 |

^A)^ Not analysed

Table S2. Kinetics data for groups of nC_5_-nC_36_ alkanes and aromatic compounds in 10 µm dispersions of Macondo oil determined by 1^st^ order rate approach with lag-phases included. Half-lives were determined from rate coefficients and lag-phases included. The goodness of fit of non-linear regressions was determined as R^2^.

| **Groups^A)^** | **Dispersion**  **(µm)** | **Lag-phase**  **(days)** | **Rate coefficient** | **Half-life**  **(days)** | **Half-life +**  **lag-phase**  **(days)** | **R^2^** |
| --- | --- | --- | --- | --- | --- | --- |
| nC5-nC9 | 10 | 0 | 0.2793 | 2.5 | 2.5 | 0.9113 |
|  | 30 | 0 | 0.2369 | 2.9 | 2.9 | 0.8360 |
| nC10-nC15 | 10 | 2.0 | 0.7718 | 0.9 | 2.9 | 0.9939 |
|  | 30 | 0.7 | 0.1566 | 4.4 | 5.1 | 0.7161 |
| nC16-nC21 | 10 | 1.9 | 0.6899 | 1.0 | 2.9 | 0.9929 |
|  | 30 | 0.7 | 0.1153 | 6.0 | 6.7 | 0.7087 |
| nC22-nC36 | 10 | 1.4 | 0.1206 | 5.7 | 7.1 | 0.9151 |
|  | 30 | 3.4 | 0.1521 | 4.6 | 8.0 | 0.7378 |
| BTEX | 10 | 7.8 | 0.3653 | 1.9 | 9.7 | 0.9992 |
|  | 30 | 10.7 | 0.2525 | 2.7 | 13.4 | 0.8929 |
| Naphthalenes | 10 | 8.0 | 0.7225 | 1.0 | 9.0 | 0.9958 |
|  | 30 | 7.3 | 0.2477 | 2.8 | 10.1 | 0.9785 |
| 2-3 ring PAH/DBT | 10 | 8.0 | 0.1603 | 4.3 | 12.3 | 0.9894 |
|  | 30 | 10.8 | 0.0813 | 8.5 | 19.3 | 0.9518 |
| 4/5 ring PAH | 10 | 7.7 | 0.1273 | 5.0 | 12.7 | 0.9571 |
|  | 30 | 1.8 | 0.0534 | 13.0 | 14.8 | 0.9647 |

^A)^ For description of aromatic compounds, see Table S1.

Table S3. Aromatic hydrocarbon target groups from GC-MS analyses of volatile and semivolatile compounds included in this study.

| **Group definition** | **Compounds** |
| --- | --- |
| BTEX | Benzene, toluene, ethylbenzene, m-xylene, p-xylene, o-xylene, |
| Naphthalenes | Naphthalene, C1-naphthalenes, C2-naphthalenes, C3-naphthalenes, C4-naphthalenes |
| 3-ring PAH/DBT | Benzo(b)thiophene, C1-benzo(b)thiophenes, C2-benzo(b)thiophene, C3-benzo(b)thiophenes, C4-benzo(b)thiophenes, C4-naphthalenes, biphenyl, acenaphthylene, acenaphthene, dibenzofuran, Fluorene, C1-fluorenes, C2-fluorenes, C3-fluorenes, phenanthrene, anthracene, C1-phenanthrenes/anthracenes, C2-phenanthrenes/anthracenes, C3-phenanthrenes/anthracenes, C4-phenanthrenes/anthracenes, dibenzothiophene, C1-dibenzothiophenes,C2-dibenzothiophenes, C3-dibenzothiophenes, C4-dibenzothiophenes |
| 4/5-ring PAH | Fluoranthene, pyrene, C1-fluoranthrenes/pyrenes, C2-fluoranthenes/pyrenes, C3-fluoranthenes/pyrenes, naphthobenzothiophene, C1-naphthobenzothiophene, C2-naphthobenzothiophene, C3-naphthobenzothiophene, C4-naphthobenzothiophene, benz(a)anthracene, chrysene, C1-chrysenes, C2-chrysenes, C3-chrysenes, C4-chrysenes, benzo(b)fluoranthene, benzo(k)fluoranthene, benzo(e)pyrene, benzo(a)pyrene, perylene, indeno(1,2,3-c,d)pyrene, dibenz(a,h)anthracene, benzo(g,h,i)perylene, retene, benzo(b)fluorene |

Fig. S1. Relative abundances of bacterial classes in seawater without oil, 10 µm dispersions and 30 µm dispersions. The results are shown in seawater used for pre-acclimation 6 days prior to start of biodegradation (Pre), and in seawater and dispersions collected at the start (0) and after 2-64 days of biodegradation.

Fig. S2. Bacterial 16S rRNA PCR-DGGE of dispersions and seawater without oil. DGGE gels of samples at the start of acclimation (pre) and during the biodegradation period are shown in A, while comparison of samples as UPGMA dendrograms are shown in B. NC is negative control.

Fig. S3. Depletion of nC_5_-nC_36_ alkanes in dispersions with unfiltered or sterilized (Ster) seawater. The depletion of SVOC n-alkanes (nC10-nC36 alkanes) was determined after normalizing targeted n-alkanes against the recalcitrant biomarker 17α(H),21β(H)-Hopane (30ab Hopane). The depletion in dispersions with unfiltered seawater is show as nonlinear decay curves, determined by 1^st^ order rates with lag-periods included. Data from non-linear regression analyses are shown in Table S3.

Fig. S4. Depletion of aromatic hydrocarbons in dispersions with unfiltered or sterilized (Ster) seawater. The depletion of SVOC compounds (naphthalenes and PAH) was determined after normalizing targeted n-alkanes against the recalcitrant biomarker 17α(H),21β(H)-Hopane (30ab Hopane). The depletion in dispersions with unfiltered seawater is show as nonlinear decay curves, determined by 1^st^ order rates with lag-periods included. Data from non-linear regression analyses are shown in Table S3.

Fig. S5. Average oil droplet size distributions generated in the oil dispersion generator at different seawater flow-rates after premixing of the oil with Corexit 9500A.


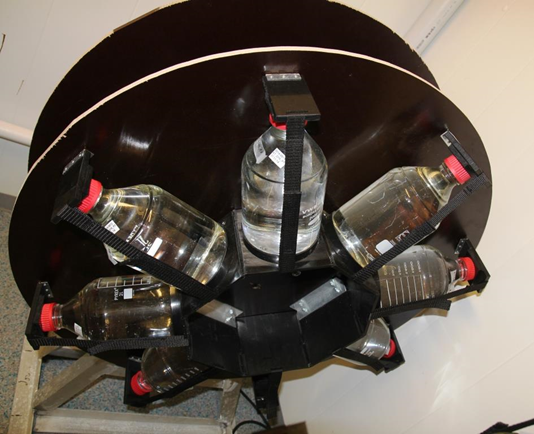


Fig. S6. Oil dispersion carousel with flasks mounted. Flasks are completely filled with oil dispersions, mounted on the carousel which is slowly rotated in clockwise direction at 0.75 r.p.m.
